# Microbiological Analysis from a Phase 2 Randomized Study in Adults Evaluating Single Oral Doses of Gepotidacin in the Treatment of Uncomplicated Urogenital Gonorrhea Caused by Neisseria gonorrhoeae

**DOI:** 10.1128/AAC.01221-18

**Published:** 2018-11-26

**Authors:** Nicole E. Scangarella-Oman, Mohammad Hossain, Paula B. Dixon, Karen Ingraham, Sharon Min, Courtney A. Tiffany, Caroline R. Perry, Aparna Raychaudhuri, Etienne F. Dumont, Jianzhong Huang, Edward W. Hook, Linda A. Miller

**Affiliations:** a GlaxoSmithKline, Upper Providence, Pennsylvania, USA; b University of Alabama at Birmingham, Birmingham, Alabama, USA

**Keywords:** *Neisseria gonorrhoeae*, microbiology, urogenital gonorrhea

## Abstract

We evaluated microbiological correlates for the successful treatment of Neisseria gonorrhoeae isolates from a phase 2 study of gepotidacin, a novel triazaacenaphthylene antibacterial, for therapy of uncomplicated urogenital gonorrhea. Culture, susceptibility testing, genotypic characterization, and frequency of resistance (FoR) were performed for selected isolates. Microbiological success was defined as culture-confirmed eradication of N. gonorrhoeae. Against 69 baseline urogenital isolates, gepotidacin MICs ranged from ≤0.06 to 1 µg/ml (MIC_90_ = 0.5 µg/ml). For gepotidacin, the ratio of the area under the free-drug concentration-time curve to the MIC (*f*AUC/MIC) was associated with therapeutic success. Success was 100% (61/61) at *f*AUC/MICs of ≥48 and decreased to 63% (5/8) for *f*AUC/MICs of ≤25. All 3 isolates from microbiological failures were ciprofloxacin resistant, had a baseline gepotidacin MIC of 1 µg/ml, and carried a preexisting ParC D86N mutation, a critical residue for gepotidacin binding. In a test-of-cure analysis, the resistance to gepotidacin emerged in 2 isolates (MICs increased ≥32-fold) with additional GyrA A92T mutations, also implicated in gepotidacin binding. Test-of-cure isolates had the same sequence type as the corresponding baseline isolates. For 5 selected baseline isolates, all carrying a ParC D86N mutation, the *in vitro* FoR to gepotidacin was low (10^−9^ to 10^−10^); the resistant mutants had the same A92T mutation as the 2 isolates in which resistance emerged. Five participants with isolates harboring the ParC D86N mutation were treatment successes. In summary, *f*AUC/MICs of ≥48 predicted 100% microbiological success, including 3 isolates with the ParC D86N mutation (*f*AUC/MICs ≥ 97). Pharmacokinetic/pharmacodynamic determinations may help to evaluate new therapies for gonorrhea; further study of gepotidacin is warranted. (This study has been registered at ClinicalTrials.gov under identifier NCT02294682.)

## Abstract (precis)

We evaluated microbiological correlates for the successful treatment of Neisseria gonorrhoeae isolates from a phase 2 study of gepotidacin, a novel triazaacenaphthylene antibacterial, for therapy of uncomplicated urogenital gonorrhea. Culture, susceptibility testing, genotypic characterization, and frequency of resistance (FoR) were performed for selected isolates.

## INTRODUCTION

The prevalence of gonorrhea infections continues to rise while effective treatment options have decreased due to progressive steadily emerging antimicrobial resistance in Neisseria gonorrhoeae (1–5 ). While investigational antibacterials have been recently evaluated in the clinic, novel antibacterials and treatment strategies are urgently needed to address the threat of potentially untreatable gonorrhea (6–9). N. gonorrhoeae gene mutations occur frequently and act through a variety of resistance mechanisms, including the alteration of antibiotic influx and efflux, enzymatic inactivation of antibacterials, and modification of antibiotic binding affinity (10, 11).

Gepotidacin (GSK2140944) is a first-in-class triazaacenaphthylene bacterial type II topoisomerase inhibitor in development for gonorrhea treatment. Gepotidacin interacts in a unique way on the GyrA subunit of bacterial DNA gyrase and the ParC subunit of bacterial topoisomerase IV, with activities against most of the target pathogens resistant to established antibacterials, including fluoroquinolones (12, 13), and against drug-resistant N. gonorrhoeae strains (14). A randomized, dose-ranging (1,500 or 3,000 mg), single oral dose phase 2 study demonstrated that for the combined dose groups, gepotidacin was 96% effective in eradicating (culture-confirmed) N. gonorrhoeae from participants with uncomplicated urogenital gonorrhea (15). Gepotidacin warrants further clinical evaluation as an alternative treatment option for gonorrhea. Our microbiological evaluation of N. gonorrhoeae isolates from this phase 2 study is presented herein.

Antibacterial therapeutic success is impacted by both the MIC of a pathogen and its exposure to an antibiotic. As such, pharmacokinetic/pharmacodynamic (PK/PD) evaluations are important to identify exposures required to maximize the efficacy against isolates with different MIC values. Adequate PK exposures may prevent the opportunities for the selection of N. gonorrhoeae mutations and the development of resistance (16–18). PK/PD indices, such as the ratio of the area under the free-drug concentration-time curve to the MIC (*f*AUC/MIC) may be used to identify optimal therapeutic regimens. Typically, nonclinical models are used to determine the PK/PD index and target that best predict a successful exposure response, which is then followed by Monte Carlo simulation using PK data to predict how many humans would likely achieve the PK/PD target for a specific dose across a clinically relevant range of MIC values (19). The PK/PD index and target may vary across clinical indications, antibacterial agents, bacterial species, and antibacterial mechanisms of action, which may be time or concentration dependent (16–18).

Because there were no validated preclinical models to determine the PK/PD target (PK/PD index and magnitude) predictive of gepotidacin efficacy for urogenital gonorrhea treatment, a nontraditional PK/PD approach was used to select gepotidacin doses for this phase 2 study. A population PK model was developed using concentration-time data from a phase 1 oral dose-ranging study in healthy volunteers (20). On the basis of nonclinical models for other bacterial species, the PK/PD index used to model theoretical efficacy was *f*AUC/MIC (21). A Monte Carlo simulation of the 1,500-mg and 3,000-mg single oral doses was then conducted to predict PK/PD magnitudes over a clinically relevant range of gepotidacin MICs. For N. gonorrhoeae isolates with a gepotidacin MIC of 1 µg/ml, the gepotidacin MIC_90_ of N. gonorrhoeae, the simulation results indicated that 90% of participants would achieve *f*AUCs of 10 and 20 µg · h/ml for the 1,500-mg and 3,000-mg doses, respectively.

We studied the microbiological data from the oral gepotidacin phase 2 urogenital gonorrhea study, including antibacterial susceptibility, quinolone resistance-determining region (QRDR) genotyping of GyrA and ParC, sequence typing, frequency of resistance (FoR), and efficacy assessments based on PK/PD magnitudes.

## RESULTS

### *In vitro* susceptibility testing.

**FIG 1:**
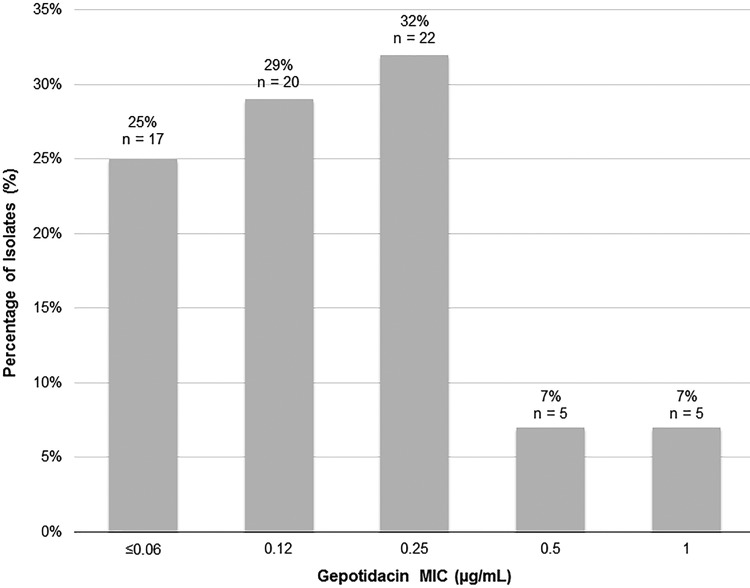
Frequency distribution of gepotidacin MICs against baseline urogenital N. gonorrhoeae isolates (*N* = 69).

Gepotidacin was active against the 69 baseline urogenital N. gonorrhoeae isolates tested with MIC values between ≤0.06 and 1 µg/ml (Fig. 1) and MIC_50_ and MIC_90_ values of 0.12 and 0.5 µg/ml, respectively (Table 1). The resistances to ciprofloxacin, penicillin, and tetracycline were 33%, 28%, and 20%, respectively, with no resistance observed for ceftriaxone, cefixime, or spectinomycin. An elevated azithromycin MIC of 2 µg/ml was observed for 2 baseline urogenital isolates.

**TABLE 1:** Antimicrobial activity of gepotidacin and comparator antimicrobial agents against urogenital *N. gonorrhoeae* isolates at baseline (*N* = 69)

Antimicrobial agent	MIC (µg/ml)			Interpretation (*n* [%])^*a*^		
	50%	90%	Range	Susceptible	Intermediate	Resistant
Gepotidacin	0.12	0.5	≤0.06 to 1	—^*b*^	—	—
Ciprofloxacin	0.004	8	≤0.001 to 16	46 (67)	0 (0)	23 (33)
Ceftriaxone	0.008	0.03	≤0.001 to 0.06	69 (100)	—	—
Cefixime	0.015	0.06	≤0.001 to 0.06	69 (100)	—	—
Azithromycin	0.25	0.5	≤0.008 to 2	—	—	—
Penicillin	0.5	4	≤0.03 to >64	1 (1)	49 (71)	19 (28)
Tetracycline	1	2	0.12 to 32	11 (16)	44 (64)	14 (20)
Spectinomycin	16	32	≤4 to 32	69 (100)	0 (0)	0 (0)

a Susceptibility interpretations were per M100-S27 Clinical and Laboratory Standards Institute breakpoints.

b —, no breakpoint was available.

Gepotidacin MICs ranged from ≤0.06 to 0.12 µg/ml and from 0.12 to 0.25 µg/ml against the 2 pharyngeal N. gonorrhoeae isolates and the 3 rectal N. gonorrhoeae isolates recovered, respectively. Resistance patterns to the tested comparator antimicrobials for these isolates were similar to those seen for the urogenital isolates.

### Microbiological response. (i) PK/PD magnitude for urogenital isolates.

The overall microbiological success was 96% (66/69) (Table 2). The PK/PD analysis showed 100% (61/61) microbiological success when the *f*AUC/MIC was ≥48, irrespective of the gepotidacin MICs of the baseline urogenital N. gonorrhoeae isolates. Among the baseline urogenital isolates with higher gepotidacin MICs, the microbiological success decreased to 63% (5/8) when the *f*AUC/MIC was ≤25. All 3 urogenital microbiological failures had *f*AUC/MICs of ≤24.

**TABLE 2:** Microbiological success by *f*AUC/MIC against urogenital *N. gonorrhoeae* at baseline

*f*AUC/MIC (range)^*a*^	*n*/*N*	Microbiological success (%)
≥198	27/27	100
95 to 103	25/25	100
48 to 49	9/9	100
24 to 25	4/6	67
12	1/2	50
Total	66/69	96

a *f*AUC/MIC, ratio of the area under the free-drug concentration-time curve to the MIC.

### (ii) Antimicrobial agent susceptibility for urogenital isolates.

For participants with baseline urogenital N. gonorrhoeae isolates with gepotidacin MICs of ≤0.5 µg/ml, the microbiological success was 100% (Table 3). Of the 5 participants with baseline urogenital N. gonorrhoeae isolates with the highest gepotidacin MIC of 1 µg/ml, 2 were microbiological successes and 3 were microbiological failures. Of the 2 participants with baseline pharyngeal N. gonorrhoeae isolates, 1 was a microbiological success (gepotidacin MIC of ≤0.06 µg/ml) and 1 was a microbiological failure (gepotidacin MIC of 0.12 µg/ml). All 3 participants with baseline rectal N. gonorrhoeae isolates (gepotidacin MIC of 0.12 µg/ml or 0.25 µg/ml) were microbiological successes.

**TABLE 3:** Microbiological response by gepotidacin MIC against *N. gonorrhoeae* at baseline^*a*^

Specimen source	Gepotidacin MIC (µg/ml)	Gepotidacin 1,500 mg (*N* = 30)				Gepotidacin 3,000 mg (*N* = 39)			
		*n*	MS (*n*)^*b*^	MF (*n*)^*c*^	Success (%)	*n*	MS (*n*)	MF (*n*)	Success (%)
Urogenital	≤0.06	8	8	0	100	9	9	0	100
	0.12	10	10	0	100	10	10	0	100
	0.25	7	7	0	100	15	15	0	100
	0.5	3	3	0	100	2	2	0	100
	1	2	1	1	50	3	1	2	33
Pharyngeal	≤0.06	0	0	0	0	1	1	0	100
	0.12	0	0	0	0	1	0	1	0
Rectal	0.12	1	1	0	100	1	1	0	100
	0.25	0	0	0	0	1	1	0	100

a Participants were only required to have a urogenital *N. gonorrhoeae* isolate at baseline to qualify for the microbiologically evaluable population. Pharyngeal or rectal *N. gonorrhoeae* isolates at baseline were not required for this population. Therefore, the *n* values for the pharyngeal and rectal data are each a subset of the full population (i.e., of the overall 69 participants in this population, 2 had a baseline pharyngeal *N. gonorrhoeae* isolate and 3 had a baseline rectal *N. gonorrhoeae* isolate).

b MS, microbiological success.

c MF, microbiological failure.

Each of the three participants classified as urogenital microbiological failures had an isolate with a gepotidacin MIC of 1 µg/ml that was also resistant to tetracycline, ciprofloxacin, and penicillin, with the exception of 1 isolate with intermediate susceptibility to penicillin. Four additional participants with baseline urogenital N. gonorrhoeae isolates that were also resistant to tetracycline and ciprofloxacin and nonsusceptible to penicillin were microbiological successes (data not shown). The 2 additional participants with baseline urogenital N. gonorrhoeae isolates with a gepotidacin MIC of 1 µg/ml that were only resistant to ciprofloxacin were microbiological successes (data not shown). No trends in microbiological outcome were observed for the few pharyngeal or rectal N. gonorrhoeae isolates with regard to baseline antimicrobial agent susceptibility.

### QRDR genotyping of N. gonorrhoeae isolates from microbiological failures.

All 3 participants who were urogenital microbiological failures had baseline N. gonorrhoeae isolates that were ciprofloxacin resistant with a baseline gepotidacin MIC of 1 µg/ml and harbored a preexisting D86N substitution due to a mutation in the *parC* gene, a critical residue in gepotidacin binding (Table 4) (12). One microbiological failure was treated with gepotidacin 1,500 mg and had a *f*AUC/MIC of 12; no change from the baseline gepotidacin MIC of 1 µg/ml was noted for the N. gonorrhoeae isolate recovered at test of cure (TOC). The other 2 microbiological failures were treated with gepotidacin 3,000 mg and had an *f*AUC/MIC of 24; a resistance to gepotidacin in both of these N. gonorrhoeae isolates was demonstrated at TOC, with gepotidacin MICs that increased ≥32-fold plus a new additional A92T substitution due to a mutation in the *gyrA* gene, which is also implicated in gepotidacin binding (12).

**TABLE 4:**
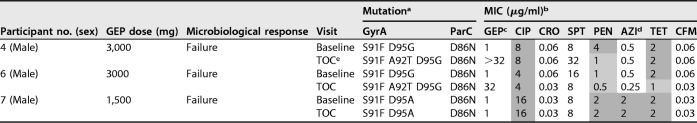


a Sequencing of quinolone resistance-determining region only.

b Dark, light, and no shading indicate resistant, intermediate, and susceptible, respectively, according to M100-S27 CLSI breakpoints. AZI, azithromycin; CFM, cefixime; CIP, ciprofloxacin; CRO, ceftriaxone; GEP, gepotidacin; PEN, penicillin; SPT, spectinomycin; TET, tetracycline.

c No breakpoints are currently available for GEP.

d The CLSI non-wild-type epidemiological cutoff value was applied.

e TOC, test of cure.

The pharyngeal microbiological failure occurred for a participant with an N. gonorrhoeae isolate that had a lower gepotidacin MIC of 0.12 µg/ml and no observed mutations in the QRDR of ParC or GyrA.

### QRDR genotyping of all N. gonorrhoeae isolates.

Of all the urogenital, pharyngeal, and rectal isolates available for sequencing, 12% (8/69) of baseline urethral isolates had a ParC D86N mutation and none of the pharyngeal or rectal isolates tested harbored a ParC D86N mutation.

All 8 isolates with the ParC D86N mutation were ciprofloxacin resistant and had 2 mutations in GyrA (Table 5). The ParC D86N mutation was observed for all 5 baseline urogenital isolates with a gepotidacin MIC of 1 µg/ml; however, this mutation was also observed for 3 isolates with lower gepotidacin MICs of ≤0.06 (1 isolate) and 0.25 µg/ml (2 isolates).

**TABLE 5:** Baseline urogenital *N. gonorrhoeae* isolates with a ParC D86N mutation

Participant no.	Mutation^*a*^		MIC (µg/ml)^*b*^								GEP dose (mg)	Microbiological response	*f*AUC/MIC^*c*^
	GyrA	ParC	GEP^*d*^	CRO	AZI	CIP	CFM	PEN	SPT	TET			
1	S91F D95G	D86N	≤0.06	0.015	0.12	2	0.03	0.25	8	0.5	1,500	Success	200
2	S91F D95A	D86N	0.25	0.008	0.25	2	0.008	0.25	16	0.5	3,000	Success	100
3	S91F D95A	D86N	0.25	0.008	0.25	2	0.015	16	32	1	3,000	Success	97
4	S91F D95G	D86N	1	0.06	0.5	8	0.06	4	8	2	3,000	Failure	24
5	S91F D95G	D86N	1	0.004	0.25	4	0.004	0.25	8	1	3,000	Success	24
6	S91F D95G	D86N	1	0.06	0.5	4	0.06	1	16	2	3,000	Failure	24
7	S91F D95A	D86N	1	0.03	2	16	0.03	2	8	2	1,500	Failure	12
8	S91F D95A	D86N	1	0.008	0.5	2	0.015	0.5	32	0.5	1,500	Success	12

a Sequencing of quinolone resistance-determining region only.

b AZI, azithromycin; CFM, cefixime; CIP, ciprofloxacin; CRO, ceftriaxone; GEP, gepotidacin; PEN, penicillin; SPT, spectinomycin; TET, tetracycline.

c *f*AUC/MIC, ratio of the area under the free-drug concentration-time curve to the MIC.

d No breakpoints are currently available for GEP.

As described above, N. gonorrhoeae isolates from all participants who were urogenital microbiological failures had gepotidacin MICs of 1 µg/ml and the ParC D86N mutation, whereas the remaining 5 isolates with the ParC D86N mutation had gepotidacin MICs of ≤0.25 µg/ml (3 isolates) and 1 µg/ml (2 isolates) and were from participants who were microbiological successes.

### Sequence types of N. gonorrhoeae isolates from microbiological failures.

Using data from a whole-genome sequencing analysis, with the primary objective of ascertaining whether a participant had failed treatment because of a new infection by a different strain, the sequence types of the baseline and TOC isolates from participants who were microbiological failures were determined by multilocus sequence typing (MLST), N. gonorrhoeae sequence typing for antimicrobial resistance (NG-STAR), and N. gonorrhoeae multiantigen sequence typing (NG-MAST) methods. With the exception of NG-STAR for participants 4 and 6 (due to the GyrA A92T mutation present in their posttreatment isolates), the results from all 3 sequence typing methods indicated that the baseline isolates from participants who were microbiological failures were the same strain as those recovered at TOC; therefore, a new infection by a different strain was unlikely to be the cause of gepotidacin treatment failure. None of the sequence typing methods alone or in combination were predictive of gepotidacin treatment failure or the emergence of resistance. The tabular results are provided in Table S1 in the supplemental material.

### Spontaneous FoR of selected N. gonorrhoeae isolates.

The 5 baseline urogenital isolates evaluated for spontaneous FoR against gepotidacin were fluoroquinolone resistant with mutations in GyrA (S91F, D95A/G) and ParC (D86N), the same mutations seen in the baseline isolates from the 3 participants who were urogenital microbiological failures. From both FoR studies, at 4× and 10× MICs, the spontaneous FoR to gepotidacin was low, at <9.1 × 10^−9^, <3.4 × 10^−9^, <3.8 × 10^−9^, ≤2.9 × 10^−9^, and <6.7 × 10^−9^ for the isolates from participants 2, 4, 5, 7, and 8, respectively. With the exception of the isolate from participant 7, no resistant mutants were isolated at 4× and 10× MICs. The urogenital baseline isolate from participant 7 had 1 resistant mutant isolated in the first study and 2 resistant mutants isolated in the second study, both at 4× the MIC of gepotidacin. These 3 isolates were genotypically characterized and found to carry a GyrA A92T mutation, the same mutation identified in the 2 isolates from participants who were urogenital failures and demonstrated resistance to gepotidacin at TOC, in addition to the preexisting GyrA S91F and D95A and ParC D86N mutations. The gepotidacin MIC increased 16-fold compared with that of the parent strain.

## DISCUSSION

Emerging N. gonorrhoeae antimicrobial resistance is an urgent public health threat. Gepotidacin, with its novel mechanism of action, warrants further study as a potential oral treatment option for gonorrhea infections. Our evaluation of microbiological data from a previously reported phase 2 study of oral gepotidacin in uncomplicated gonorrhea (15) provides insights for further evaluation, including antimicrobial susceptibility, QRDR mutations, sequence types, FoR, and efficacy assessments based on *f*AUC/MIC magnitudes. Our evaluation of *f*AUC/MIC magnitudes and their associated treatment outcomes represents a novel approach for predicting gonorrhea therapy outcomes.

When doses were selected for this clinical study, gepotidacin had been evaluated using an *in vivo* mouse gonorrhea vaginal colonization model, which provided potential efficacy information but was not validated for PK/PD characterization (22). Therefore, a nonclinical model utilized for other bacterial species was adapted to determine that gepotidacin efficacy is concentration dependent and that, by extension, *f*AUC/MIC may be the most appropriate PK/PD index for predicting gepotidacin efficacy (21). A population PK model with phase 1 data and a Monte Carlo simulation was used for exposure predictions. While the optimum PK/PD index and target for gepotidacin efficacy against N. gonorrhoeae were not known, the simulations predicted that 90% of participants would achieve *f*AUCs of 10 and 20 µg · h/ml for the 1,500- and 3,000-mg doses, respectively. The PK predictions for dose selection were close to the actual study results, where *f*AUCs were approximately 12 and 24 µg · h/ml for the low and high doses, respectively. In addition, the distribution of gepotidacin MICs for N. gonorrhoeae in this study was similar to that from a prior surveillance study, where approximately 7% of N. gonorrhoeae isolates had a gepotidacin MIC of 1 µg/ml (23). Progress continues to be made in mouse models for gonorrhea infection, which will also continue to advance preclinical research efforts (24).

Microbiological success was achieved for all participants with an *f*AUC/MIC of ≥48, including success for 3 participants with urogenital N. gonorrhoeae isolates with a ParC D86N mutation and an *f*AUC/MIC of ≥97. All 3 participants who were classified as microbiological urogenital failures had an *f*AUC/MIC of ≤24.

N. gonorrhoeae isolates from participants who were urogenital failures had common features. All were ciprofloxacin resistant, had a baseline gepotidacin MIC of 1 µg/ml, and harbored a preexisting ParC D86N mutation, which has a prevalence of up to 30% in fluoroquinolone-resistant N. gonorrhoeae (4, 25). In addition, resistance emerged in 2 of these isolates (≥32-fold increase in the gepotidacin MIC from baseline to TOC), with an additional GyrA A92T mutation in the TOC isolate. Three sequence typing methods indicated that the baseline isolates from participants who were microbiological failures were the same strain as those recovered at TOC; therefore, a new infection by a different strain was unlikely the cause of treatment failure. Both ParC D86 and GyrA A92 are critical for the interaction of gepotidacin with bacterial type IIA topoisomerases (12). As gepotidacin works by inhibiting both DNA gyrase and topoisomerase IV, the preexisting ParC D86N mutation in these treatment-failure isolates reduced the dual-targeting activity of gepotidacin to a single target and increased the resistance emergence potential. The preexisting ParC D86N mutation appeared to be recessive and did not significantly affect the initial gepotidacin MIC. However, this mutation likely increased the potential for resistance development and contributed to the large gepotidacin MIC increase seen at TOC with the additional target mutation, which was observed for 2 N. gonorrhoeae isolates from participants who were microbiological failures. This is further supported by subsequent whole-genome sequencing of a subset of N. gonorrhoeae isolates, which did not identify target mutations in GyrA/GryB, ParC/ParE, or in other proteins encoded by genes associated with gepotidacin failure or resistance emergence (data not shown). These data suggest that future doses of gepotidacin need to achieve a higher PK/PD magnitude to support optimum gepotidacin efficacy in gonorrhea treatment, especially in global regions where N. gonorrhoeae isolates with higher gepotidacin MICs and fluoroquinolone resistance rates may be observed (5). The highest daily oral dose of gepotidacin that has be studied in the clinic is 6,000 mg (26, 27).

The FoR to gepotidacin at 4× and 10× MICs for a selected subset of isolates carrying the same mutations as the baseline isolates from participants who were urogenital microbiological failures was low (10^−9^ to 10^−10^). The 3 resistant mutants recovered in the FoR studies were found to carry an additional GyrA A92T mutation, which was also found in 2 urogenital TOC isolates in which resistance emerged. The increase in gepotidacin MIC (16-fold) seen for the FoR mutants was similar to the increase (≥32-fold) seen for the 2 urogenital TOC isolates in which resistance emerged. These results suggest that these clinical isolates containing mutations in GyrA (S91F, D95A/G) and ParC (D86N) show a low *in vitro* frequency of spontaneous resistance to gepotidacin and that although mutants with an additional GyrA A92T mutation were isolated, the *in vitro* frequency of this occurrence was also low. The reason these low *in vitro* FoR results are discordant with the amount of resistance emergence seen in the clinical trial (2 of 69 participants) is unknown.

Previous FoR studies with N. gonorrhoeae isolates also showed a low FoR (10^−9^ to 10^−10^) to gepotidacin at 4× and 8× MICs for fluoroquinolone-susceptible and -resistant isolates, and no resistant mutants were recovered (14), suggesting that gepotidacin exhibits well-balanced dual targeting for DNA gyrase and topoisomerase IV. However, none of the isolates tested in this prior study had a preexisting ParC D86N mutation (14). The microbiological data from the present clinical trial are largely consistent with previous *in vitro* FoR results (14), in that N. gonorrhoeae isolates lacking the preexisting ParC D86N mutation did not develop resistance to gepotidacin.

There were several limitations of this microbiological evaluation. Only a few N. gonorrhoeae isolates were from extragenital body sites. Because antimicrobial concentrations may vary at these mucosal sites, future studies should seek to include participants with extragenital N. gonorrhoeae infections. The N. gonorrhoeae isolates from this study only reflect U.S. epidemiology, and different resistance patterns may be observed in other geographic regions. However, in a recent publication, gepotidacin had an MIC_90_ of 1 µg/ml against a global collection of 252 N. gonorrhoeae isolates (28), which was similar to the gepotidacin MIC_90_ of 0.5 µg/ml in this clinical study. Potential studies for future consideration are whole-genome sequencing of additional N. gonorrhoeae isolates recovered from the study and experiments to understand the potential for the transmission of ParC D86N and GyrA A92T mutations to susceptible gonococcal strains.

Our data demonstrated that microbiological success was achieved for all participants with urogenital N. gonorrhoeae isolates when the *f*AUC/MIC was ≥48. Microbiological success decreased to 63% (5/8) when *f*AUC/MICs were ≤25, resulting in urogenital microbiological failure for 3 participants with ciprofloxacin-resistant N. gonorrhoeae isolates, which had a baseline gepotidacin MIC of 1 µg/ml and harbored a preexisting ParC D86N mutation, thereby reducing the activity of gepotidacin for 1 of the 2 bacterial targets. However, microbiological success was achieved for 5 participants with isolates that harbored a preexisting ParC D86N mutation, 2 with an *f*AUC/MIC ≤24, and 3 with a higher *f*AUC/MIC of ≥97. These results indicate that further evaluation of gepotidacin in the treatment of gonorrhea is warranted, including a demonstration that higher exposures increase the efficacy and suppress the resistance in key isolate subsets.

## MATERIALS AND METHODS

### Study design.

The phase 2, randomized, multicenter, open-label, dose-ranging single oral dose study of gepotidacin (1,500 mg or 3,000 mg in a 1:1 ratio stratified by sex) for the treatment of urogenital gonorrhea was described previously (15). Briefly, a single oral dose of gepotidacin was administered at baseline, followed by a test-of-cure (TOC) analysis 3 to 7 days after dosing. Pretreatment and TOC urogenital swab specimens were obtained; rectal and pharyngeal specimens were also collected. The microbiologically evaluable population consisted of 69 randomly assigned participants (67 male and 2 female) with culture-confirmed urogenital gonorrhea at baseline who received gepotidacin and returned for TOC (15). Of the 69 microbiologically evaluable participants, 2 also had culture-confirmed pharyngeal gonorrhea and 3 also had culture-confirmed rectal gonorrhea (15). Microbiological success was defined as culture-confirmed eradication of N. gonorrhoeae at TOC. Microbiological failure was defined as culture-confirmed bacterial persistence of N. gonorrhoeae at TOC or the inability to determine the response (e.g., lost sample) of the baseline pathogen at TOC.

The guidelines of the International Council for Harmonisation of Technical Requirements for Pharmaceuticals for Human Use, good clinical practice guidelines, and applicable country-specific requirements were followed, including institutional review board approval at each study site. All participants provided signed informed consent.

### Microbiological evaluation.

Specimens were processed for culture at local laboratories according to accepted microbiological procedures (29). All presumptively identified N. gonorrhoeae isolates from local laboratories were sent to the central laboratory for confirmatory identification testing (University of Alabama at Birmingham, Birmingham, AL). Agar dilution antimicrobial susceptibility testing was performed according to Clinical and Laboratory Standards Institute (CLSI) and Gonococcal Isolate Surveillance Program methods at the central laboratory (30–32). MICs were determined for gepotidacin, azithromycin, cefixime, ceftriaxone, ciprofloxacin, penicillin G, spectinomycin, and tetracycline, and where available, CLSI breakpoints were applied. For azithromycin, CLSI epidemiological cutoff values were applied (31).

QRDR genotyping of GyrA and ParC was performed for all N. gonorrhoeae isolates by GlaxoSmithKline (Collegeville, PA). N. gonorrhoeae was subcultured from a frozen stock onto chocolate II agar plates and incubated at 35°C for 18 h with 5% carbon dioxide. A loopful (1-µl loop) of cells from a fresh plate was transferred to 50 µl Tris-EDTA buffer (pH 8) and boiled for 10 min. The tube was then put on ice for 2 min and centrifuged at 14,000 rpm in an Eppendorf 5415 C for 1 min. The supernatant (2 µl) was used as the template for PCR. The PCR primers for the amplification of N. gonorrhoeae *gyrA* and *parC* encoding gyrase subunit A and topoisomerase IV subunit C, respectively, were described previously by Vernel-Pauillac et al. (33). PCR was carried out using a GeneAmp PCR system 9700 under the following conditions: 5 min at 95°C; 35 cycles of 30 s at 95°C, 45 s at 48°C, and 1 min at 72°C; 7 min at 72°C for 1 cycle, and then 4°C. Invitrogen PCR SuperMix High Fidelity (10790-020; Invitrogen) was used. The PCR products were separated, visualized, sized by electrophoresis on a 1% agarose gel containing ethidium bromide, and purified with a QIAquick PCR purification kit (28104; Qiagen) according to the manufacturer’s instructions. The PCR products were sequenced with a BigDye Terminator v3.1 cycle sequencing kit and analyzed with a 3730xl DNA analyzer; all equipment were from Applied Biosystems (Foster City, CA). Isolate sequences were compared to the parent and reference FA1090 sequences obtained from The National Center for Biotechnology Information (https://www.ncbi.nlm.nih.gov). Lasergene SeqMan software (DYNASTAR, Inc., Madison, WI) was used to identify nucleotide changes resulting in amino acid residue substitutions.

Whole-genome sequencing was conducted for the baseline and TOC isolates from all participants who were microbiological failures. The FASTA sequences for each isolate were entered into the following online sequence typing databases: MLST (www.mlst.net), NG-STAR (https://ngstar.canada.ca), and NG-MAST (www.ng-mast.net).

To assess FoR, 2 separate studies were performed by GlaxoSmithKline (Collegeville, PA) using Oxoid GC agar base containing BB BBL IsovitaleX enrichment in study 1 and Remel agar base plus hemoglobin and BB BBL IsovitaleX Enrichment in study 2. A select set of N. gonorrhoeae isolates recovered from participants, including microbiological successes and failures, from the phase 2 clinical trial having the GyrA (S91F and D95A/G) and ParC (D86N) mutations were tested. This study was performed to determine the FoR to gepotidacin in baseline urogenital isolates carrying the mutations associated with microbiological failure.

Gepotidacin was added to the appropriate medium-containing molten agar to yield 20 ml of agar at the correct multiple of the MIC for each organism. Plates containing gepotidacin at 4× MIC or 10× MIC were poured and left to cool and to solidify. Plates containing no compound were also prepared to obtain viable counts and to serve as a growth control. Cultures were prepared by direct colony suspension in saline solutions to a turbidity equivalent to a 4 McFarland standard prepared from colonies grown on a chocolate plate after an overnight incubation at 35°C in 5% carbon dioxide. To determine the number of CFU present in the initial test inoculum, each suspension was serially diluted 1:10, and three 20-µL drops from each dilution were plated on agar and incubated overnight at 35°C in 5% carbon dioxide. Counting was performed at the dilution that provided distinguishable colonies, and an average from the 3 samples was used to calculate the number of CFU in the original suspension. In addition, 100 µl of each cell suspension was spread on the surfaces of plates containing the appropriate multiple of the MIC of the compound and on a control agar plate containing no compound. The plates were incubated at 35°C in 5% carbon dioxide_._ After 48 h of incubation, single colonies that grew on the FoR plates were streaked onto new plates containing identical drug concentrations and incubated at 35°C in 5% carbon dioxide (30, 31). These purified resistant colonies were then isolated on plain chocolate agar plates and frozen in broth medium containing glycerol. To confirm their resistance phenotype, the susceptibility of these isolates to gepotidacin was tested by agar dilution methodology. PCR and sequencing of the QRDR were performed on all resistant isolates.

FoR was calculated by dividing the number of confirmed resistant colonies growing on antibiotic-containing plates by the total number of CFU in the initial test inoculum. Colonies were defined as resistant if their MICs were ≥4× the MIC of the parent strain.

### PK.

The *f*AUC of gepotidacin achieved over 24 h for each gepotidacin dose was estimated from data of healthy volunteers in a previously described population PK 2-compartment model with an absorption lag time and zero-order input for oral absorption (20). The model had a low coefficient of variation for all model parameter estimates of ≤2.2 and a residual variability of 39%. The variance was not inflated, as PK variability was expected to be similar between participants with gonorrhea and healthy volunteers. The mean gepotidacin 2-h postdose plasma concentrations in participants with gonorrhea were 2.89 and 6.35 µg/ml for the 1,500-mg and 3,000-mg doses, respectively (15).

## Supplementary Material

Supplemental file 1

## References

[1] Centers for Disease Control and Prevention. 2017 Reported STDs in the United States, 2016. High burden of STDs threaten millions of Americans. Centers for Disease Control and Prevention, Atlanta, GA. www.cdc.gov/nchhstp/newsroom/docs/factsheets/STD-Trends-508.pdf.

[2] Centers for Disease Control and Prevention. 2017 Sexually transmitted diseases surveillance 2016. Centers for Disease Control and Prevention, Atlanta, GA. www.cdc.gov/std/stats16/CDC_2016_STDS_Report-for508WebSep21_2017_1644.pdf.

[3] European Centre for Disease Prevention and Control. 2016 Annual epidemiological report 2016. Gonorrhea. European Centre for Disease Prevention and Control, Stockholm, Sweden ecdc.europa.eu/sites/portal/files/documents/Gonorrhoea%20AER_0.pdf.

[4] Unemo M, Shafer WM 2014 Antimicrobial resistance in *Neisseria gonorrhoeae* in the 21st century: past, evolution, and future. Clin Microbiol Rev 27:587–613. doi:10.1128/CMR.00010-14. 24982323 PMC4135894

[5] Wi T, Lahra MM, Ndowa F, Bala M, Dillon JR, Ramon-Pardo P, Eremin SR, Bolan G, Unemo M 2017 Antimicrobial resistance in *Neisseria gonorrhoeae*: global surveillance and a call for international collaborative action. PLoS Med 14:e1002344. doi:10.1371/journal.pmed.1002344. 28686231 PMC5501266

[6] Alirol E, Wi TE, Bala M, Bazzo ML, Chen X-S, Deal C, Dillon JR, Kularatne R, Heim J, Hooft van Huijsduijnen R, Hook EW, Lahra MM, Lewis DA, Ndowa F, Shafer WM, Tayler L, Workowski K, Unemo M, Balasegaram M 2017 Multidrug-resistant gonorrhea: a research and development roadmap to discover new medicines. PLoS Med 14:e1002366. doi:10.1371/journal.pmed.1002366. 28746372 PMC5528252

[7] Centers for Disease Control and Prevention. 2013 Gonorrhea treatment guidelines: revised guidelines to preserve last effective treatment option. Centers for Disease Control and Prevention, Atlanta, GA. www.cdc.gov/std/treatment/2010/gonorrhea-treatment-guidelines-factsheet.pdf.

[8] World Health Organization. 2017 Global priority list of antibiotic resistant bacteria to guide research, discovery, and development of new antibiotics. World Health Organization, Geneva, Switzerland. www.who.int/medicines/publications/WHO-PPL-Short_Summary_25Feb-ET_NM_WHO.pdf?ua=1.

[9] Unemo M, Bradshaw CS, Hocking JS, de Vries HJC, Francis SC, Mabey D, Marrazzo JM, Sonder GJB, Schwebke JR, Hoornenborg E, Peeling RW, Philip SS, Low N, Fairley CK 2017 Sexually transmitted infections: challenges ahead. Lancet Infect Dis 17:e235–e279. doi:10.1016/S1473-3099(17)30310-9. 28701272

[10] Suay-García B, Pérez-Gracia MT 2017 Drug-resistant *Neisseria gonorrhoeae*: latest developments. Eur J Clin Microbiol Infect Dis 36:1065–1071. doi:10.1007/s10096-017-2931-x. 28210887

[11] Unemo M, del Rio C, Shafer WM 2016 Antimicrobial resistance expressed by *Neisseria gonorrhoeae*: a major global public health problem in the 21st century. Microbiol Spectr 4:ei10-0009-2015. doi:10.1128/microbiolspec.EI10-0009-2015. PMC4920088 27337478

[12] Bax BD, Chan PF, Eggleston DS, Fosberry A, Gentry DR, Gorrec F, Giordano I, Hann MM, Hennessy A, Hibbs M, Huang J, Jones E, Jones J, Brown KK, Lewis CJ, May EW, Saunders MR, Singh O, Spitzfaden CE, Shen C, Shillings A, Theobald AJ, Wohlkonig A, Pearson ND, Gwynn MN 2010 Type IIA topoisomerase inhibition by a new class of antibacterial agents. Nature 466:935–940. doi:10.1038/nature09197. 20686482

[13] Biedenbach DJ, Bouchillon SK, Hackel M, Miller LA, Scangarella-Oman NE, Jakielaszek C, Sahm DF 2016 *In vitro* activity of gepotidacin, a novel triazaacenaphthylene bacterial topoisomerase inhibitor, against a broad spectrum of bacterial pathogens. Antimicrob Agents Chemother 60:1918–1923. doi:10.1128/AAC.02820-15. 26729499 PMC4776004

[14] Farrell DJ, Sader HS, Rhomberg PR, Scangarella-Oman NE, Flamm RK 2017 *In vitro* activity of gepotidacin (GSK2140944) against *Neisseria gonorrhoeae*. Antimicrob Agents Chemother 61:e02047-16. doi:10.1128/AAC.02047-16. 28069643 PMC5328517

[15] Taylor SN, Morris DH, Avery AK, Workowski KA, Batteiger BE, Tiffany CA, Perry CR, Raychaudhuri A, Scangarella-Oman NE, Hossain M, Dumont EF 2018 Gepotidacin for the treatment of uncomplicated urogenital gonorrhea: a phase 2 dose-ranging, single-oral dose evaluation. Clin Infect Dis 67:504–512. doi:10.1093/cid/ciy145. 29617982 PMC6070052

[16] Ambrose PG, Bhavnani SM, Rubino CM, Louie A, Gumbo T, Forrest A, Drusano GL 2007 Pharmacokinetics-pharmacodynamics of antimicrobial therapy: it’s not just for mice anymore. Clin Infect Dis 44:79–86. doi:10.1086/510079. 17143821

[17] Bulik CC, Bader JC, Zhang L, Van Wart SA, Rubino CM, Bhavnani SM, Sweeney KL, Ambrose PG 2017 PK-PD compass: bridging infectious diseases pharmacometrics to the patient’s bedside. J Pharmacokinet Pharmacodyn 44:161–177. doi:10.1007/s10928-017-9518-0. 28353185

[18] Nielsen EI, Cars O, Friberg LE 2011 Pharmacokinetic/pharmacodynamic (PK/PD) indices of antibiotics predicted by a semimechanistic PKPD model: a step toward model-based dose optimization. Antimicrob Agents Chemother 55:4619–4630. doi:10.1128/AAC.00182-11. 21807983 PMC3186970

[19] Clinical and Laboratory Standards Institute. 2016 Development of *in vitro* susceptibility testing criteria and quality control parameters, 4th ed Document M23 Clinical and Laboratory Standards Institute, Wayne, PA.

[20] Hossain M, Tiffany CA, Day LA, Dumont EF 2013 Population pharmacokinetic modeling of first-time-in-human data of GSK2140944, a novel antimicrobial compound. Abstr 53rd Intersci Conf Antimicrob Agents and Chemother, abstr F-1217.

[21] Bulik CC, Okusanya ÓO, Lakota EA, Forrest A, Bhavnani SM, Hoover JL, Andes DR, Ambrose PG 2017 Pharmacokinetic-pharmacodynamic evaluation of gepotidacin against Gram-positive organisms using data from murine infection models. Antimicrob Agents Chemother 61:e00115-16. doi:10.1128/AAC.00115-16. PMC5278741 27872075

[22] Jerse AE, Wu H, Packiam M, Vonck RA, Begum AA, Garvin LE 2011 Estradiol-treated female mice as surrogate hosts for *Neisseria gonorrhoeae* genital tract infections. Front Microbiol 2:107. doi:10.3389/fmicb.2011.00107. 21747807 PMC3129519

[23] Scangarella-Oman NE, Dixon P, Koeth LM, DiFranco-Fisher J, Miller LA 2016 Analysis of agar dilution MIC testing methods and variables and in vitro activity of gepotidacin (GSK2140944) against Neisseria gonorrhoeae, poster Sunday-462. ASM Microbe, Boston, MA, 16 to 20 June 2016.

[24] Rice PA, Shafer WM, Ram S, Jerse AE 2017 *Neisseria gonorrhoeae*: drug resistance, mouse models, and vaccine development. Annu Rev Microbiol 71:665–686. doi:10.1146/annurev-micro-090816-093530. 28886683

[25] Dewi BE, Akira S, Hayashi H, Ba-Thein W 2004 High occurrence of simultaneous mutations in target enzymes and MtrRCDE efflux system in quinolone-resistant *Neisseria gonorrhoeae*. Sex Transm Dis 31:353–359. doi:10.1097/00007435-200406000-00007. 15167645

[26] O’Riordan W, Tiffany C, Scangarella-Oman N, Perry C, Hossain M, Ashton T, Dumont E 2017 Efficacy, safety, and tolerability of gepotidacin (GSK2140944) in the treatment of patients with suspected or confirmed gram-positive acute bacterial skin and skin structure infections. Antimicrob Agents Chemother 61:e02095-16. doi:10.1128/AAC.02095-16. 28373199 PMC5444153

[27] Hossain M, Tiffany CA, McDonald M, Dumont EF 2014 Safety and pharmacokinetic of repeat escalating oral doses of GSK2140944, a novel bacterial topoisomerase inhibitor, abstr 2320. Abstr 54th Intersci Conf Antimicrob Agents and Chemother.

[28] Jacobsson S, Golparian D, Scangarella-Oman N, Unemo M 2018 *In vitro* activity of the novel triazaacenaphthylene gepotidacin (GSK2140944) against MDR *Neisseria gonorrhoeae*. J Antimicrob Chemother 73:2072–2077. doi:10.1093/jac/dky162. 29796611 PMC6927889

[29] Garcia LS, Isenberg HD (ed). 2010 Clinical microbiology procedures handbook, 3rd ed ASM Press, Washington, DC.

[30] Clinical and Laboratory Standards Institute. 2015 Methods for dilution antimicrobial susceptibility tests for bacteria that grow aerobically; approved standard, 10th ed Document M07-A10 Clinical and Laboratory Standards Institute, Wayne, PA.

[31] Clinical and Laboratory Standards Institute. 2017 Performance standards for antimicrobial susceptibility testing, 27th ed Document M100-S27 Clinical and Laboratory Standards Institute, Wayne, PA.

[32] Gonococcal Isolate Surveillance Program. 2016 Protocol. Centers for Disease Control and Prevention, Atlanta, GA. www.cdc.gov/std/gisp/GISP-Protocol-May-2016.pdf.

[33] Vernel-Pauillac F, Hogan TR, Tapsall JW, Goarant C 2009 Quinolone resistance in *Neisseria gonorrhoeae*: rapid genotyping of quinolone resistance-determining regions in *gyrA* and *parC* genes by melting curve analysis predicts susceptibility. Antimicrob Agents Chemother 53:1264–1267. doi:10.1128/AAC.01104-08. 19124663 PMC2650556

