# Supplementary material for: Microbiological Analysis from a Phase 2 Randomized Study in Adults Evaluating Single Oral Doses of Gepotidacin in the Treatment of Uncomplicated Urogenital Gonorrhea Caused by Neisseria gonorrhoeae
Source: Antimicrob Agents Chemother. 2018 Nov 26;62(12):e01221-18. doi: 10.1128/AAC.01221-18 (PMC6256812; doi:10.1128/AAC.01221-18)
Supplement: Supplemental file 1 [file zac012187642s1.pdf]

SUPPLEMENTAL MATERIALS

Title: **Microbiological Analysis From a Phase 2 Randomized Study in Adults Evaluating Single Oral Doses of Gepotidacin in the Treatment of Uncomplicated Urogenital Gonorrhea Caused by *Neisseria gonorrhoeae***

Authors: Nicole E. Scangarella-Oman, Mohammad Hossain, Paula B. Dixon, Karen Ingraham, Sharon Min, Courtney A. Tiffany, Caroline R. Perry, Aparna Raychaudhuri, Etienne F. Dumont, Jianzhong Huang, Edward W. Hook III, Linda A. Miller

TABLE S1 Sequence typing results for select *Neisseria gonorrhoeae* isolates

Participant number (Gender)	Specimen source	Visit	Sequence typing method		
			MLST	NG-STAR	NG-MAST
4 (Male)	Urethral	Baseline	7827	38	13246
		Test-of-cure	7827	NA*	13246
6 (Male)	Urethral	Baseline	7827	38	2318
		Test-of-cure	7827	NA*	2318
7 (Male)	Urethral	Baseline	9363	168	12302
		Test-of-cure	9363	168	12302
9 (Male)	Pharyngeal	Baseline	9363	NA	unknown
		Test-of-cure	9363	NA	unknown

* Unable to assign the score due to the acquisition of the GyrA A92T mutation.
MLST, Multi Locus Sequence Typing; NA, not available due to no defined sequence type found in the database; NG-MAST, *N. gonorrhoeae* Multi Antigen Sequence Typing; NG-STAR, *N. gonorrhoeae* Sequence Typing for Antimicrobial Resistance.
